# Multiple Bone Metastasis of Sclerosing Epithelioid Fibrosarcoma 12 Years after Initial Surgery—Increasing Ki-67 Labeling Index

**DOI:** 10.1155/2009/953750

**Published:** 2009-03-29

**Authors:** Atsuko Kanno, Masahito Hatori, Masami Hosaka, Koshi N. Kishimoto, Munenori Watanuki, Mika Watanabe, Eiji Itoi

**Affiliations:** ^1^Department of Orthopaedic Surgery, Tohoku University School of Medicine, Sendai 980-8574, Japan; ^2^Department of Pathology, Tohoku University School of Medicine, Sendai 980-8574, Japan

## Abstract

Sclerosing epithelioid fibrosarcoma (SEF) is a rare sarcoma of low-grade malignancy. There has been no report to describe the comparison of histological features of SEF between primary and metastatic lesions in spite of high local recurrence rate. We report the histological changes and increasing Ki-67 labeling index of the primary and metastatic lesions of SEF. The patient was a 31-year-old man. At 18, a tumor in the abdominal wall was excised. At 23, the tumor recurred which was removed again. At 30, he was referred to our hospital because of swelling and pain in the chest. Histological examination of the chest wall tumor showed epithelioid cells arranged like alveolar pattern with dense collagen stroma. These findings were consistent with those of SEF. Abdominal and the rib tumors showed the same immunohistochemistrical expression. It is noteworthy that the tumor cells of the rib lesion showed increased cellularity, and its Ki-67 activity was higher as compared with the abdominal tumor, suggestive of progression of malignancy of SEF.

## 1. Introduction

Sclerosing epithelioid fibrosarcoma (SEF) is a very rare sarcoma which occurs in the deep musculature. Extensive English literature survey reveals that there have been only fifteen reports so far. There is wide age of spectrum and median age is 45 and equal sex distribution [1].

It is first reported by Meis-Kindblom in 1995. The author described SEF as a variant of fibrosarcoma simulating carcinoma. This tumor is characterized by epithelioid cells arranged in nest and strands in highly sclerosing matrix. SEF is a relatively low-grade fibrosarcoma histologically [2].

On the other hand, high rate of recurrence and metastasis has been reported. Meis-Kindblom et al. reported that local recurrence occurred in 53% and metastasis occurred in 43%. Their interval of metastasis was 4.7 to 14 years. In some other cases, uncontrollable recurrence and metastasis occurred after uneventful some years. They stated that SEF is a clinically malignant sarcoma [3–5].

There is no report about the comparison of histological characteristics of SEF between primary and metastatic lesions in spite of high local recurrence rate. In some case reports, the biopsy from the metastasis lesion was performed, and the pathology was consistent with the primary lesion [4–7]. However, there was no detailed description about the grade of malignancy change except one report [6]. We report an SEF patient, who had metastasis to the bones twelve years after the initial operation, and the metastatic lesion of SEF showed higher grade of malignancy than the primary lesion.

## 2. Case Report

**Figure 1:**
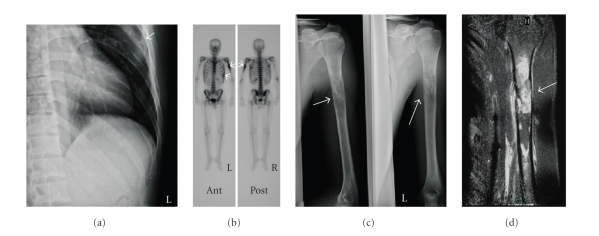
(a) Plain radiogram showing bone destruction of the rib (arrow). (b) Bone scintigram showing increased spots in the bilateral humerus and the left rib (arrow). (c) Plain radiogram of the left humerus showing an osteolytic lesion (arrow). (d) T1 weighted magnetic resonance imaging showing gadolinium enhancement (arrow).

A 30-year-old man presented with 2-year history of bump and pain in his left chest. In 1995, the soft tissue tumor of abdominal wall was excised. In 2000, the recurrent tumor was excised again. In January of 2007, he was referred to our hospital. There was tenderness in the left chest. Radiological examination revealed a lesion with bone destruction in the left 6th rib (Figure 1(a)). CT revealed a pleural lesion. Bone scintigraphy and positron emission tomography (PET) showed increased uptake in the bilateral humeral shaft and left rib (Figure 1(b)). Plain X-ray films demonstrated an osteolytic lesion in the humeral shaft (Figure 1(c)). The cortex was thinned. MRI revealed the lesion in the left humeral shaft with low signal intensities on T1 weighted images and iso to high signal intensities on T2 weighted images. The mass was enhanced after gadolinium injection (Figure 1(d)).

**Figure 2:**
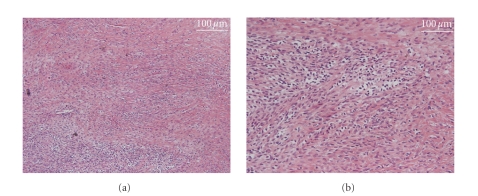
the specimen of the abdominal tumor excised in 1995 showing abundant collagen proliferation (a) and alveolar pattern (b).

Retrospective histological examination of the specimen removed in 1995 revealed that the lesion was composed of many spindle cells admixed with abundant collagen matrix (Figure 2(a)). Epithelioid cells arranged in an alveolar pattern (Figure 2(b)). Mitosis was conspicuous. It was consistent with SEF.

**Figure 3:**
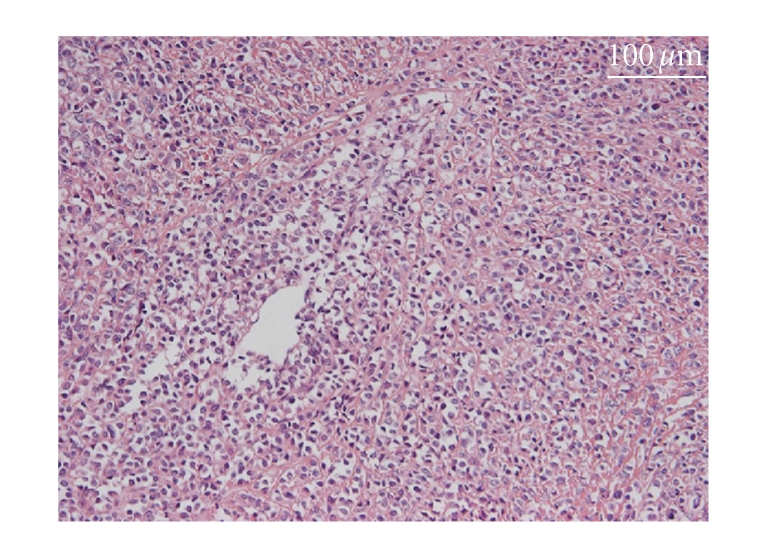
The specimen of the abdominal tumor excised in 2000 showing epithelioid cells arranged in an alveolar pattern, too.

The tumor removed from abdominal wall in 2000. There was richness in abundant collagen. The features of the tumor were similar to those of the previous surgery specimen. However, this tumor showed increased cellularity. In this specimen, epithelioid cells were more dominant than spindle cells, and the epithelioid cells were arranged in alveolar pattern (Figure 3). These cells showed nuclear polymorphism, and increased frequency of mitosis 15 cells per 10 high-power fields (HPF). Necrosis was found in the specimen. This area revealed ghost-like figures. These features well corresponded to SEF showing higher-grade malignancy compared to the primary lesion.

**Figure 4:**
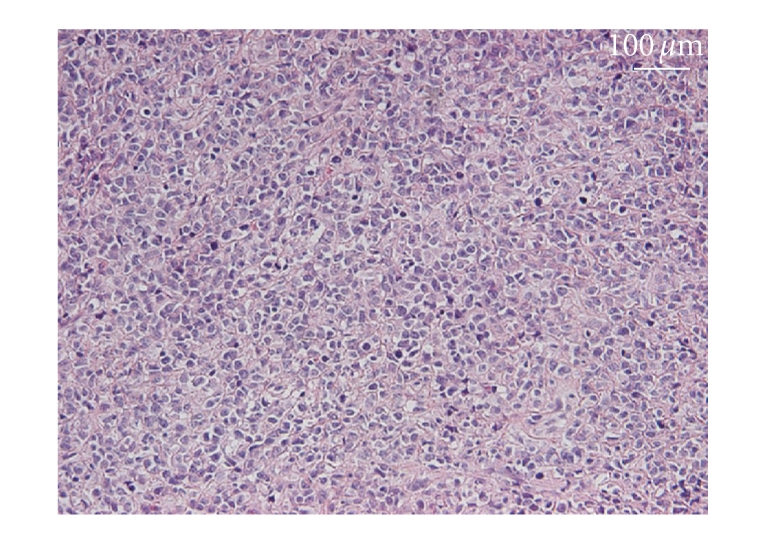
The specimen of the rib excised in 2007 showing cellular atypia and prominent nuclear pleomorphism.

To confirm the diagnosis, excisional biopsy of the 6th rib was performed. Macroscopically, the bone marrow was occupied by a white solid tumor. Microscopically, the specimen was composed of dysplastic cells in the hyalinized stroma. These cells were arranged in epithelioid and alveolar patterns too. In contrast to the primary lesion, this lesion was characterized by much higher cellularity, high nuclear/cell rate, and more prominent nuclear polymorphism (Figure 4). Mitosis was more prominent with 30 to 40 cells per 10 HPF. Necrosis was frequently found in the lesion. Ghost-like figures were also present too.

Immunohistochemical examination revealed vimentin-positive cells. Although it was focal, tumor cells were positive for HHF-35. CD99 had a weak immunostaining pattern. Tumor cells were negative for *α*-SMA, desmin, CD34, S-100 protein, cytokeratin (AE1/AE3), and epithelial membrane antigen (EMA). All three specimens showed same features by immunohistochemistry.

**Figure 5:**
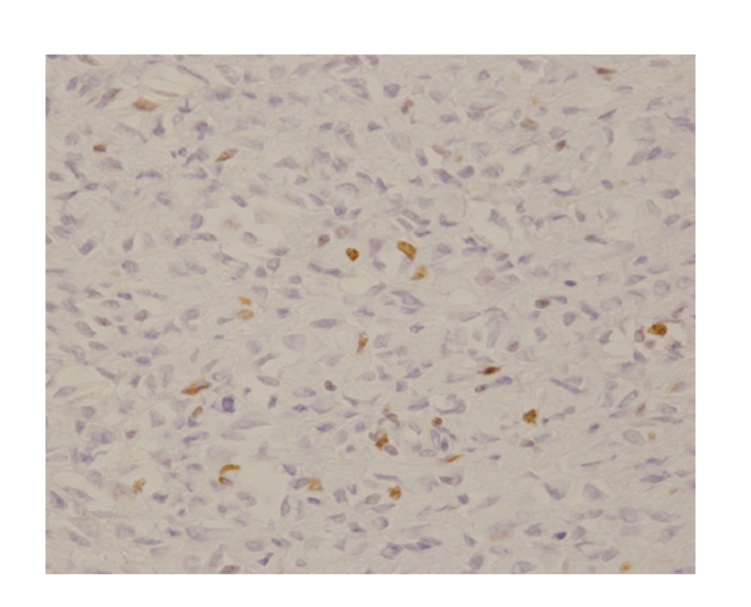
Ki-67 staining of the specimen resected in 1995. There are few Ki-67 positive cells. Ki-67 labeling index was 7 to 8%.

**Figure 6:**
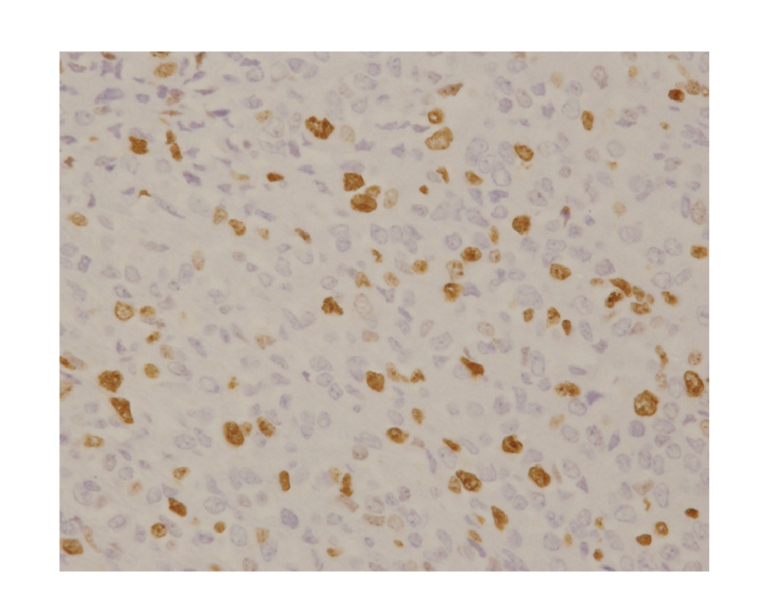
The specimen resected in 2000. There were more Ki-67 positive cells than Figure 5. Ki-67 labeling index was 20%.

**Figure 7:**
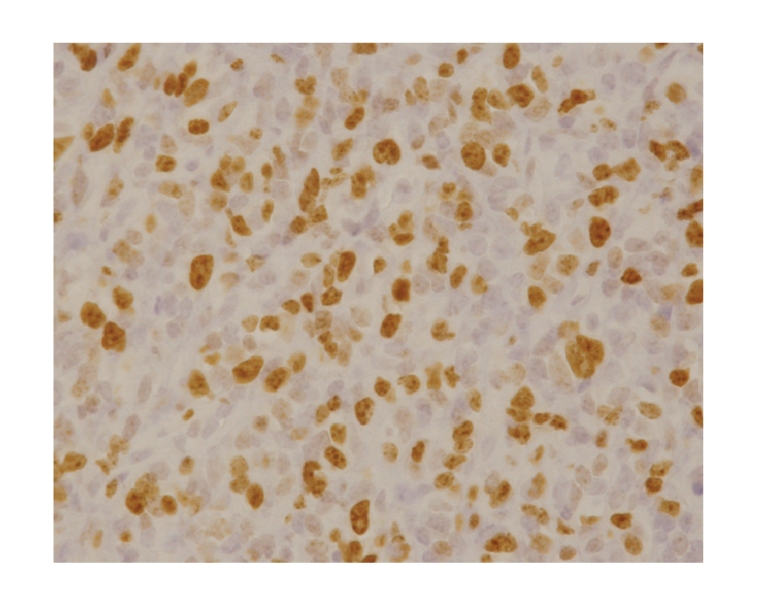
The specimen resected in 2007. More cells were stained by Ki-67. Ki-67 labeling index was 60%.

Ki-67 labeling indices were 7∼8%, 20%, and 60% in the tumor excised in 1995, in 2000, and in 2007, respectively, (Figures 5, 6, and 7). In conclusion, the primary lesion was SEF and that the lesion of the rib was bone metastasis from SEF in the abdomen.

In February 2007, pathological fracture occurred in the left humeral shaft. Since it was a metastatic lesion, chemotherapy using ifosfamide and adriamycin was performed. However, this chemotherapy was discontinued because of acute myocardial infarction. Since the tumor size is increasing, the patient was treated by radiation therapy to the left humerus. After that, he hoped the second opinion, so he was referred to another hospital.

## 3. Discussion

### 3.1. Histology of the SEF

SEF is characterized by uniform, small, and round to ovoid epithelioid cells with clear cytoplasm arranged in distinct nest and cords, embedded in a hyalinized fibrous stroma. This histology stimulates infiltrating carcinoma. In contrast, there are hypocellular and fibromatous areas which contain spindle cells and abundant collagen, simulating fibroma [2]. There is the morphologic variance in the sclerosing matrix. Antonescu et al. also pointed out this, so the author described that “*there was a patchwork of zones of varying size, shape, and cellularity*” in the SEF [3]. Eyden et al. described that all the SEF showed variable cellularity in five cases [8]. In some other reports variable cellularity was described [9, 10]. Hemorrhage and necrosis were also occasionally seen [2, 6–8, 11].

In addition, the SEF is characterized by a range of other morphologic appearance. Meis-Kindblom et al. described that there were myxoid zones with cyst formation in the SEF and that more cellular myxoid zones stimulated myxoid fibrosarcoma [2]. Antonescu et al. also reported that poorly delineated myxoid area were present in four cases [3]. In some reports there were fibromatous zones which resemble classic fibrosarcoma [2, 3, 12, 13]. In addition, the staghorn vessels, which were hemangiopericytoma pattern in some reports [7, 11]. So, there are many differential diagnoses: nodular fasciitis, desmoid, infiltrating carcinoma, monophasic synovial sarcoma, clear cell sarcoma, hemangioperiendothelioma, sclerosing lymphoma, myxoid fibrosarcoma, and osteosarcoma.

In our case, polymorphic epithelioid cells arrange in nest and strand, and there is much abundant collagen and fibroblasts. There is nuclear polymorphism too. It is consistent with Meis-Kindblom's report [2]. In addition, histological analysis showed that mitosis and necrosis area were much more frequently seen in the rib tumor than the abdominal wall tumors resected in 1995 and in 2000. Cellularity increased, which strongly suggests increasing malignancy.

### 3.2. Immunohistochemical Feature

Meis-Kindblom described that in most fibrosarcoma, immunohistochemical staining was of limited value although it was useful for excluding other lesions [2]. From past available reports described in English [2–16], the only consistent finding is diffuse reactivity for vimentin. Some cases express NSE [2, 6] and S-100 protein [2, 12, 14] although they were weak or focal. In some cases, tumor cells were positive for EMA, although most of them were focal positive [2, 6, 10, 13, 14].

In the cases which were difficult to diagnose, ultrastructural examination was performed and the features displayed the features of fibroblasts [2–8, 10, 11, 14].

In our case, vimentin was diffusely positive. Cytokeratin and *α*-SMA were negative. Carcinoma, synovial sarcoma, and epithelioid sarcoma were ruled out, and, CD34 was negative, so hemangiopericytoma is less likely. Moreover, three specimens (tumor resected in 1995, 2000, and 2007) showed same feature by immunohistochemistry. So, we concluded that these tumors are the same.

### 3.3. Proliferation Marker

Ki-67 is a nuclear antigen which is expressed in late G1, S, M, G2 growth phases. In other words, Ki-67 is expressed only when mitosis occur. There is a correlation between high Ki-67 labeling index, especially, more than 20%, and poor clinical diagnosis in soft tissue sarcoma [17]. In some cases of SEF Ki-67 labeling index is performed [2, 7, 10, 11, 13], Ki-67 labeling index was 1 to 5%. It is consistent of low mitotic rate, which were 0 to 10 mitoses per 10 high-power fields. On the other hand, high rate of local recurrence and distant metastasis are reported. Meis-Kindblom concluded that neither proliferation markers nor mitotic rate correlated with prognosis [2].

In our case, Ki-67 labeling index has changed among three specimens. The abdominal tumor resected in 1995 showed 7 to 8% labeling index. The abdominal tumor resected in 2000 showed 20%, the bone tumor of the rib showed 60% Ki-67 labeling index, respectively. We concluded that malignancy grade has increased.

### 3.4. Clinical Feature

Clinically, in many cases of SEF local recurrence [2–4, 7, 8, 12] and distant metastasis occurred [2–8, 14]. Especially, uncontrollable local recurrence and distant metastasis occur after the uneventful years. It is not uncommon that metastasis occurs some years after primary surgery [2, 3, 5, 14]. Meis-Kindblom reported that eight patients had local recurrence in 15 patients and that the median interval to the first local recurrence was 4.8 years (2.3 to 11 years). The author also reported that six patients had distant metastasis in 14 patients whose data were available and that the median interval to metastases was 7.7 years (4.7 to 14 years) [2].

In our case, metastasis has occurred after 12 years. This feature is consistent with these reports.
